# Dopamine-induced changes to thalamic GABA concentration in impulsive Parkinson disease patients

**DOI:** 10.1038/s41531-022-00298-8

**Published:** 2022-04-05

**Authors:** Paula Trujillo, Alexander K. Song, Kaitlyn R. Hay, Megan Aumann, Yan Yan, Hakmook Kang, Manus J. Donahue, Daniel O. Claassen

**Affiliations:** 1 Neurology, Vanderbilt University Medical Center, Nashville, TN USA; 2 Biostatistics, Vanderbilt University Medical Center, Nashville, TN USA; 3 Psychiatry and Behavioral Sciences, Vanderbilt University Medical Center, Nashville, TN USA

**Keywords:** Diagnostic markers, Parkinson's disease

## Abstract

Impulsivity is inherent to behavioral disorders such as substance abuse and binge eating. While the role of dopamine in impulse behavior is well established, γ-aminobutyric acid (GABA) therapies have promise for the treatment of maladaptive behaviors. In Parkinson disease (PD), dopaminergic therapies can result in the development of impulsive and compulsive behaviors, and this clinical syndrome shares similar pathophysiology to that seen in addiction, substance abuse, and binge-eating disorders. We hypothesized that impulsive PD patients have a reduced thalamic GABAergic response to dopamine therapy. To test this hypothesis, we employed GABA magnetic resonance spectroscopy, D2-like receptor PET imaging, and clinical and quantitative measures of impulsivity in PD patients (*n* = 33), before and after dopamine agonist administration. We find a blunted thalamic GABA response to dopamine agonists in patients with elevated impulsivity (*p* = 0.027). These results emphasize how dopamine treatment differentially augments thalamic GABA concentrations, which may modify behavioral impulsivity.

## Introduction

Therapies that modulate γ-aminobutyric acid (GABA) have been assessed in addiction and mood disorders, and are a promising treatment option^1^, especially for use in alcohol dependence^2^ substance abuse, and bipolar disorder. Commonly studied medications include baclofen, a GABA_B_ agonist, and anti-epileptic treatments (e.g., topiramate)^3^. While the localization for where these treatments exert their effect is unknown, it is well-appreciated that altered mesolimbic and mesocortical networks subserve maladaptive behaviors. Impulsivity is often discussed in the context of the cognitive process that underlies these behavioral manifestations^4–7^, and is closely linked to reward-based decision making. Reward responsivity is modulated by the mesolimbic dopamine pathway, especially dopamine release in the ventral striatum. Understanding how this increase can be augmented through interventions that target GABA requires advancements in methods that can assess the neurobiology of GABA, dopamine, impulsivity, and human behavior.

In Parkinson disease (PD), 15–40% of patients treated with dopamine medications can develop maladaptive behaviors^8,9^ that are characterized by compulsive participation in naturally occurring rewards, commonly referred to as impulsive and compulsive behaviors (ICB)^10,11^. This clinical syndrome is of important translational relevance to the study of dopamine and reward-based decision making^9,12^, as behaviors emerge as a result of dopamine treatments for motor symptoms^13–15^. PD patients with ICBs compulsively engage in reward-based activities that frequently include increased time spent on hobbies, compulsive eating, gambling, spending, and/or hypersexuality^16–18^. Dopamine agonists (DAA) therapies induce greater risk-taking in ICB patients^19,20^ and while the prevalence is uncommon with L-DOPA monotherapy^18,21^, when treatment doses are reduced, or discontinued, patients often note the resolution of the behavioral symptom.

In this context, the study of ICBs can provide important advances in allowing for translational studies that assess GABAergic neurotransmitter response to dopamine treatments, and their relevance to behavioral symptoms in humans. DAA preferentially target D3 and D2 receptors (D2-like receptors), with less affinity to D1^22^. This class of medications is one of the strongest risk factors for developing ICBs, not only in PD but also in other disorders such as restless leg syndrome^23^. The nature of this medication’s side effects, the use of these medications in routine practice, and willingness for patients to withhold medications over an extended period of time, provide a unique opportunity to devise studies that assess neurobiological effects of medication use. These studies have expanded our understanding that DAA augments metabolic activity in ventral striatum circuitry^24^, and modify reward-based circuits that connect mesolimbic and mesocortical regions^25,26^.

The effect of dopamine therapies on thalamic function and associated networks has not been a consistent focus of research. Thalamocortical networks are largely modulated by excitatory/inhibitory outputs arising from the globus pallidus and subthalamic nucleus, but emerging data suggest dopamine itself may regulate this network through concerted action on thalamic-based dopamine receptors^27,28^. The main neurochemical regulator of thalamocortical inhibition is GABA, and several studies point to thalamic GABA dysfunction in PD^29–31^.

We hypothesized that patients with ICB will demonstrate a blunted thalamic GABAergic response to DAA treatment and a net-excitatory effect from direct-pathway stimulation on the thalamocortical network. In this study, a within-patient design allowed us to assess the effect of DAA medication on thalamic GABA in PD patients manifesting with ICBs, and determine how thalamic D2-like receptors modulate this effect. For this purpose, we employed J-edited magnetic resonance spectroscopy (MRS), which enables simultaneous quantification of GABA and glutamate (as Glx, a complex of glutamate and glutamine) in humans non-invasively in vivo. Finally, we conducted an exploratory analysis to evaluate if the medication effect on thalamic GABA was mediated by thalamic D2-like dopamine receptor availability, as measured by PET imaging with [^18^F]-Fallypride, a high-affinity D2-like receptor ligand that can measure D2/3 non-displaceable binding potential (BP_ND_).

## Results

### Participants

All participants provided informed, written consent for this prospective study. This study enrolled 33 participants with PD (19 males, median age = 64 years; range = 45–80 years), who underwent neuroimaging, cognitive, and neurological assessments before and after DAA administration. Nineteen patients had a clinical diagnosis of active ICB symptoms (ICB+), based on clinical evaluation. The predominant behavior was compulsive eating in 16 participants, with the other 3 participants noting hypersexual behavior in addition to compulsive eating. Demographic and clinical features are presented in Table 1. All participants completed two MRI sessions: following withdrawal from their dopaminergic medication (Off-DAA), and in their optimal state of DAA therapy (On-DAA).

**Table 1:** Demographic and clinical data.

	PD ICB−	PD ICB+	*p-*value
*N*	14	19	
Sex (M/F)	9/5	10/9	0.754
Age (years)	67.2 ± 7.4	61.6 ± 6.5	0.039
Disease duration (years)	7.2 ± 4.2	4.3 ± 3.3	0.021
Movement Disorders Society-United Parkinson Disease Rating Scale (MDS-UPDRS)			
Part II	18.1 ± 10.3	18.9 ± 11.0	0.867
Part III (Off)	30.7 ± 10.6	29.5 ± 14.2	0.572
Part III (On)	27.8 ± 10.6	20.0 ± 9.9	0.031
Questionnaire for impulsive Compulsive Disorders in Parkinson’s disease Rating Scale (QUIP-RS)	17.4 ± 9.3	29.1 ± 13.3	0.008
Montreal Cognitive Assessment (MoCA)	25.8 ± 2.3	27.0 ± 2.1	0.07
Center for Epidemiologic Studies Depression Scale (CESD-R)	12.2 ± 6.4	17.1 ± 10.0	0.08
Dopamine replacement therapy			
Total levodopa daily dose (LEDD) (mg/day)	792.2 ± 436.1	642.7 ± 366.9	0.414
Dopamine agonist single dose equivalent (mg/day)	102.0 ± 109.0	118.0 ± 64.0	0.126

Data are reported at mean ± standard deviation. *P*-values are from the Wilcoxon rank-sum test for continuous variables and the Chi-squared test for categorical variables.

### GABAergic response to dopamine agonist

**Fig. 1:**
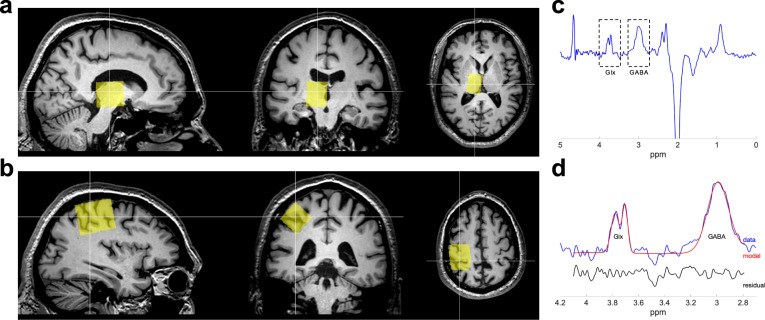
MRS voxel placement and a representative spectrum. Location of the MRS voxels in **a** thalamic and **b** motor cortex areas. **c** Example of the edited spectrum. **d** Representative fitting of the GABA+ peak at 3 ppm and the Glx peak at 3.75 ppm in the edited spectrum; acquired data are in blue, fit in red, and residual in black.

**Fig. 2:**
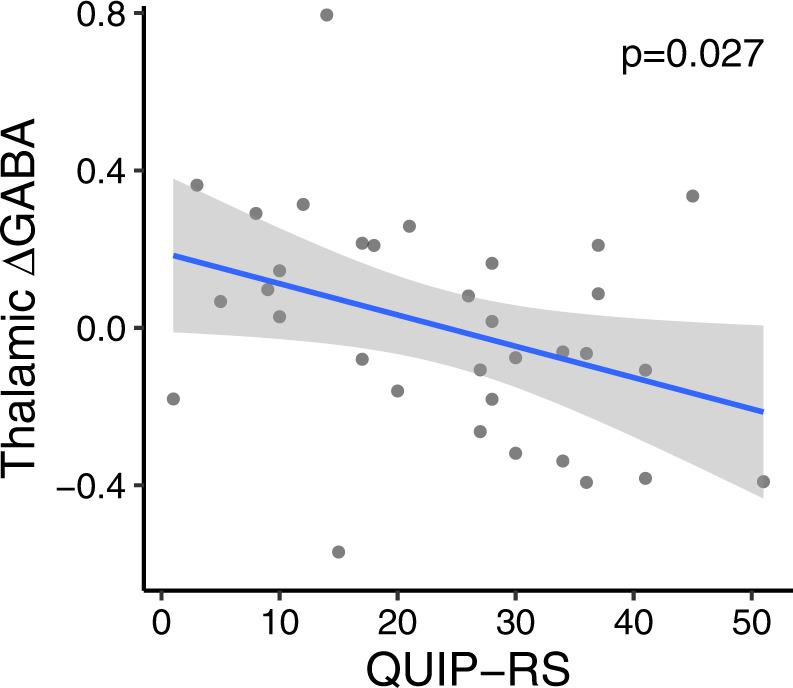
Relationship between GABAergic response to dopamine agonist therapy and impulsivity. Partial correlation between the pharmacological effect of dopamine agonist (DAA) on thalamic GABA (ΔGABA) and the Questionnaire for Impulsive-Compulsive Disorders in Parkinson’s Disease-Rating Scale (QUIP-RS) score. ΔGABA values are adjusted for age and DAA dosage. The QUIP-RS score is a clinical measure of ICB symptom severity, in which higher values represent the predilection towards reward-based behaviors. The regression analysis showed that higher QUIP-RS scores were associated with lower thalamic ΔGABA (*p* = 0.027), while adjusting for age and DAA dosage.

J-edited MRS was used to non-invasively quantify GABA and glutamate concentration separately in the thalamus and motor cortex before and after DAA medication administration. Figure 1 shows voxel placement and a representative spectrum. Thalamic GABAergic response to DAA (estimated as ΔGABA = [GABA_ON_ – GABA_OFF_]/GABA_OFF_, where GABA_ON,OFF_ represent the thalamic GABA+ estimate in the On- and Off-DAA conditions) was significantly blunted for ICB+ vs. ICB− (*p* = 0.045) while accounting for age and DAA dosage, but this difference was not significant for the motor cortex GABA (*p* = 0.486). Moreover, thalamic ΔGABA was significantly correlated to self-reported ratings of impulsivity as defined by the Questionnaire for Impulsive-Compulsive Disorders in Parkinson’s Disease-Rating Scale (QUIP-RS). Here, lower thalamic changes in GABA correlated with greater impulsivity (adjusted for age and DAA dosage) (Fig. 2) (*p* = 0.027). No relationship was observed between ΔGABA in the motor cortex and impulsivity (*p* = 0.216). The thalamic glutamatergic response (measured using the Glx levels) to DAA was not significantly different between ICB+ and ICB− (*p* = 0.305), and it was not associated with QUIP scores (*p* = 0.389).

### Association between thalamic GABAergic response to dopamine agonist and dopamine receptor availability

**Fig. 3:**
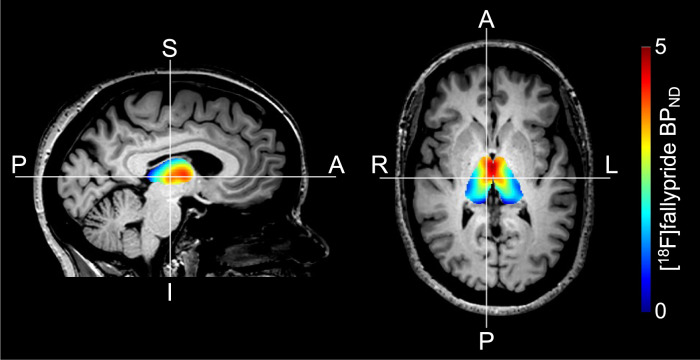
Thalamic [^18^F]fallypride BP_ND_. Representative example of [^18^F]fallypride BP_ND_ values in the thalamus overlaid on a T1-weighted image.

**Fig. 4:**
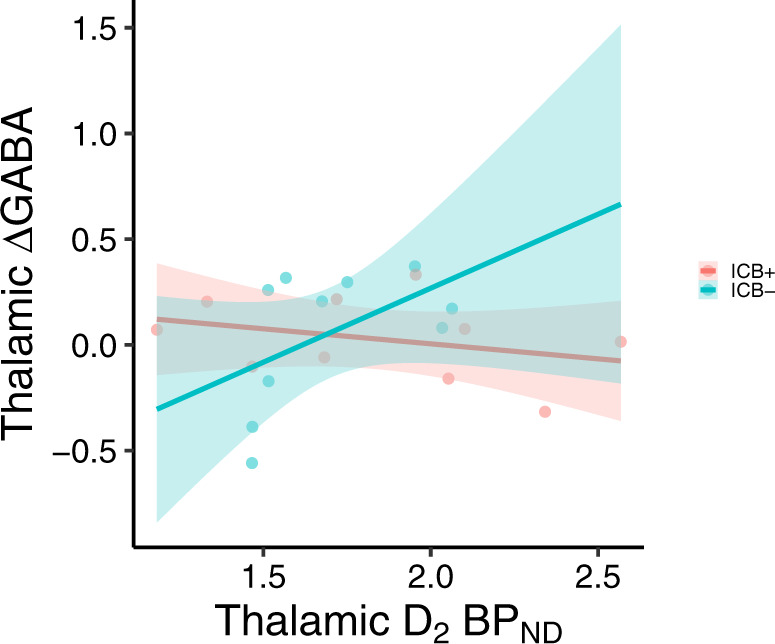
Association between GABAergic response to dopamine agonist therapy and thalamic dopamine receptor availability. Scatter plot showing the relationship between the thalamic GABAergic response to DAA medication (ΔGABA) and thalamic [^18^F]fallypride BP_ND_ in ICB+ and ICB− patients, adjusting for age. Dark center lines indicate fitted lines, while lighter colored bands denote confidence bands. This relationship appears to differ between ICB+ and ICB− patients (GLM: Thalamic ΔGABA ~ thalamic BP_ND_ + ICD status + thalamic BP_ND_*ICD status + age, *p*-value for the interaction term thalamic BP_ND_*ICD status: *p* = 0.051).

Figure 3 shows an example of the [^18^F]fallypride BP_ND_ values in the thalamus. Across all participants, we did not observe an association between thalamic ΔGABA and D2-like receptor availability, while adjusting for age (*p* = 0.973). However, we observed that this relationship appears to differ between ICB+ and ICB− patients (*p* = 0.051) (Fig. 4).

## Discussion

Interrogating the effect of dopamine on the direct and indirect pathway in humans has generally been difficult, owing to a lack of ability to measure GABA concentration, limitations in isolating and interpreting network-based activation of dopamine receptor subtypes, and inability to understand concerted effects of these manipulations on human behavior. Neurotransmitter investigations are frequently conducted under pharmacological manipulation in sedated animals^32,33^, or with tissue biopsy or autopsy procedures, with the obvious caveat that in vitro chemical profiles may differ from their in vivo counterparts^34^, and anesthesia may confound neurotransmitter measurements. This study is an important translational extension of decades of research assessing dopamine effects on reward and behavior, as we are able to measure the effect of dopamine receptor agonists on in vivo thalamic GABA concentrations in humans using MRS^35–37^.

Our results support a clear link between patient-reported assessments of reward responsiveness, the clinical-defined severity of compulsive reward-driven behaviors in the determination of ICB (compulsive eating and hypersexuality, emerging in time after the initiation therapy for motor symptoms of PD), and thalamic GABA response to DAA. If the effect of GABA at the thalamic level is indeed linked to behavioral symptoms of compulsive reward-based behaviors, future treatments that can focus on localized modification of GABA, based on clinical and radiologic data, may allow for improved target validation, patient identification, and therapeutic effect.

Dopamine modulates basal ganglia circuitry through the direct and indirect pathways, and while conventional models reference the motor effects of these pathways, new models incorporate effects on decision making and behavior^38^. In rodents, direct pathway stimulation results in compulsive reward-driven behavior, and indirect pathway stimulation results in more flexible decision making^39^. Common to both pathways is thalamocortical circuitry, where stimulation of the direct pathway (D1 receptors) results in reduced thalamic inhibition of excitatory thalamocortical signaling, while stimulation of the indirect pathway (D2-like receptors) results in inhibition of this pathway^40,41^. Dopamine signaling plays an important role in behavioral regulation and decision making^42–44^, and dopamine-induced changes to the balance of the direct and indirect activation may provide a biological basis for medication effects that manifest with impulsive and compulsive reward-based behaviors.

Previous studies of compulsive eating and hypersexuality have linked thalamic signaling to behavioral manifestations of appetitive motivation. For instance, differential GABA agonism in the paraventricular nucleus or medial dorsal nucleus of the thalamus can increase or suppress appetite^45^. Likewise, thalamic activation in response to sexual stimuli is greater in patients with hypersexual behavior^46^. We interpret the difference in DAA-induced thalamic GABA to emphasize a biological effect of this medication effect on the balance of the direct and indirect pathway activation. These pathways operate in a concert given their opposing effect on movement and behavior. The relative absence of a thalamic GABA response in patients with ICB suggests a greater direct pathway stimulation or blunted indirect pathway activation. Previous studies that employ optogenetic techniques in assessments of rodent behavior indicate that activation of direct pathway neurons in the ventral striatum (i.e., nucleus accumbens) induce a behavior similar to what is encountered in patients with ICBs^39^. These compulsive or persistent pursuits are of naturally rewarding behaviors (e.g., shopping, gambling, eating, and sex). We cannot rule out the possibility that the medication effect acts on thalamic dopamine receptors. We have previously described reduced D2-like receptors in the thalamus of PD patients^47^, but have not seen differences in patients with and without ICBs^48^. D2-like receptors in the thalamus are highly concentrated in the medial dorsal nucleus, and anterior nucleus^49^ (Fig. 3). As only a subset of the participants in the study (*n* = 20) underwent the PET scans, these results should be interpreted carefully with the expectation that a larger sample size may capture relationships not currently evident. These nuclei are important in modulating decision making and behavior, thus we speculate that the GABAergic response is localized here, hence the relation to behavioral symptoms in patients. The limitation of MRS is that we cannot provide accurate localization to specific nuclei of the thalamus. Furthermore, the lack of change in motor cortex GABA^50^, suggests that the medication effect may be localized subcortically, where future studies should assess striatal GABA changes in response to dopamine therapies.

In summary, these data provide insights into the effect of dopamine therapy on non-dopamine-related neurotransmission, namely GABA concentration. The findings of altered response based on clinical presentation and patient-reported outcomes measure are important translational efforts linking dopamine effects on reward, basal ganglia, and thalamocortical circuitry. These data have potential relevance not only to PD but also to studies of addiction science.

## Methods

### Participants

Patients with idiopathic PD meeting UK Brain Bank criteria^51^ treated with DAA therapy were recruited from the Movement Disorders Clinic at Vanderbilt University Medical Center. The study has been carried out in accordance with The Declaration of Helsinki, and all subjects provided written, informed consent before participating in the study in compliance with the standards of ethical conduct in human investigation regulated by the Vanderbilt Institutional Review Board.

PD severity was assessed by a board-certified neurologist using the Movement Disorders Society-Unified Parkinson’s Disease Rating Scale (MDS-UPDRS) parts II and III. These scales assess the self-reported quality of life and motor symptom severity, respectively^52^. The cognitive screening was performed using the Montreal Cognitive Assessment (MoCA) to exclude patients with frank dementia^53^, requiring a score of at least 22, and depression symptoms were screened using the Center for Epidemiologic Studies Depression Scale Revised (CESD-R)^54^. Patients’ current prescribed dosages of dopaminergic medication, including Levodopa and DAA, were converted to levodopa equivalent daily dose (LEDD) using the conversion factors and formulae reported in ref. ^55^. Patients were excluded if they had (i) history of neurological diseases other than PD, (ii) clinical symptoms of dementia, depression, cerebrovascular, or cardiovascular disease, (iii) an implanted deep brain stimulator, or (iv) implanted hardware that was contraindicated for 3 T magnetic resonance imaging (MRI).

Patients were categorized as current (or active) ICB+ or ICB− based on a detailed semi-structured behavioral interview with the patient and spouse. This interview evaluated the presence of compulsive behaviors with onset following DAA administration, with specific attention towards the previously reported categories of compulsive shopping, eating, hypersexuality, gambling, and hobbyism^56–58^. Prior to the interview, participants completed two self-report scales: Questionnaire for Impulsive-Compulsive Disorders in Parkinson’s Disease-Rating Scale (QUIP-RS)^58^, and Barrett Impulsiveness Scale (BIS-11)^59^. Patients were designated as ICB+ if the criteria for present ICBs as defined in the Diagnostic and Statistical Manual of Mental Disorders (DSM-IV-TR)^60^ had emerged following the initiation of DAA.

All patients enrolled in the study completed two MRI sessions. One session was performed following withdrawal from their dopaminergic medication (Off-DAA), and the other was when patients were in their optimal state of DAA therapy (On-DAA). In the Off-DAA condition, patients refrained from all dopaminergic medications for a total time of 5-half lives of DAA. Practically, this was at least 36 h for DAA, and 16 h for Levodopa due to differences in pharmacokinetic properties^61,62^. This period was deemed sufficient to eliminate DAA effects, while minimizing potential patient discomfort^63^. In the On-DAA state, patients were evaluated after taking their prescribed DAA medication, having withheld Levodopa for at least 16 h. Extended-release DAA compounds (taken by 12 patients) were administered 6 h before MR scanning, whereas non-extended release DAA (taken by 18 patients) were administered 2 h before scanning. No changes in medication dosages or addition or discontinuation of either drug for clinical purposes were made at any time during study participation.

### MRS acquisition

Patients were scanned in the Off- and On-DAA states using a 3 T MRI scanner (Achieva, Philips Healthcare, the Netherlands) with body coil transmit and 32-channel head coil reception. Scanning included a 3D structural *T*_*1*_-weighted whole-brain image, (MPRAGE, TR/TE = 8.9/4.6 ms; turbo gradient echo factor = 131; spatial resolution = 1 × 1 × 1 mm^3^), and single-voxel J-edited MRS using MEGA-PRESS^64^ (TR/TE = 3000/68 ms; 320 transients; 2048 data points at a spectra width of 2 kHz). The spectroscopy voxels were planned off orthogonal reconstructions of the high-resolution *T*_1_-weighted scan and placed in the right thalamic area (voxel dimensions = 30 × 22 × 28 mm^3^) (Fig. 1a) and the right motor cortex (voxel dimensions = 40 × 25 × 25 mm^3^) (Fig. 1b). Editing pulses (14 ms, 140 Hz bandwidth) were applied at 1.9 ppm and 8 ppm on alternate scans. An unedited MRS scan without water suppression was also acquired for normalization.

GABA concentration in the motor cortex was also analyzed as a control region, as we hypothesize that DAA will have a regional effect in thalamic GABA but will not affect GABA concentration in the motor cortex. In addition, we investigated the effect of DAA on thalamic excitatory activity by measuring thalamic glutamate concentration.

### MRS data analysis

MRS analysis was performed using Gannet 3.0^65^. Frequency and phase correction and outlier rejection was applied (Fig. 1c). The single GABA+ peak at 3 ppm (GABA peak with a contribution from macromolecule signals) was fitted using a Gaussian model (Fig. 1d), and the levels of GABA+ were calculated from the area under the peak. The unsuppressed water spectrum was processed to obtain the area under the water peak, which was then used to estimate GABA+ concentration relative to water. The levels of glutamate were obtained from the Glx peak (a complex of glutamate and glutamine) at 3.75 ppm (Fig. 1d), which was fitted to a Gaussian model to obtain the area under the peak, and then normalized by the area under the water peak.

To account for the underlying tissue composition, we applied the α-correction^66,67^. GannetCoRegister was used to register the MRS voxel to the *T*_*1*_-weighted image, and tissue segmentation was performed by merging the results obtained from FSL FAST and FSL FIRST (Supplementary Fig. 1) (FSL v5.0.2.1, FMRIB, Oxford, UK). The MRS voxel mask was then applied to the tissue segmentation to determine the tissue voxel fractions for gray matter (GM), white matter (WM), and cerebrospinal fluid (CSF) (Supplementary Fig. 1). The compartment correction (using *α* = 0.5, Wanasapura values for relaxation parameters, and 36.1, 43.3, and 53.8 mol/dm^3^ for MR-visible water concentrations for WM, GM, and CSF, respectively), and tissue normalization were applied to account for differences in GABA+/water concentration between GM and WM, and to obtain the compartment corrected thalamic and motor cortex GABA+ concentration^67^. A similar correction was applied to the Glx/water measures to obtain the compartment corrected Glx concentration.

### PET acquisition and processing

A subset of 20 participants (10 ICB+ and 10 ICB−) completed a PET scan with [^18^F]Fallypride as described in refs. ^47,48^. D2-like receptor levels were estimated using the simplified reference tissue model (SRTM) performed in PMOD software version 3.7 (PMOD Technologies, Zurich Switzerland) to measure [^18^F]fallypride binding potential (BP_ND_; the ratio of specifically bound [^18^F]fallypride to its nondisplaceable concentration as defined under equilibrium conditions)^47,48^. BP_ND_ images were co-registered to the T1-weighted image using FSL FLIRT (FSL v5.0.2.1, FMRIB, Oxford, UK). FSL FIRST was used to obtain the thalamic mask and the mean BP_ND_ values were recorded (Fig. 3).

### Statistical analysis

The effect of DAAs on thalamic GABA was estimated as ΔGABA = [GABA_ON_ – GABA_OFF_]/GABA_OFF_, where GABA_ON,OFF_ represent the thalamic GABA+ estimate in the On- and Off-DAA conditions. To understand whether thalamic GABA changes are different between ICB+ and ICB−, we performed a general linear regression model (GLM) analysis specifying thalamic ΔGABA as dependent variable, ICB status as an independent variable, and age and DAA dosage (i.e., agonist single dose equivalent) as covariates (GLM: Thalamic ΔGABA ~ ICD status + age + DAA dosage). To evaluate if thalamic GABA changes were related to a quantitative marker of impulsivity, we performed GLM analyses specifying ΔGABA as the dependent variable, QUIP-RS score as an independent variable, and age and DAA dosage as covariates (GLM: Thalamic ΔGABA ~ QUIP-RS + age + DAA dosage). The above GLM analyses were also performed for the motor cortex GABA and the thalamic glutamate as dependent variables. Finally, we tested the association between the changes in thalamic GABA and the thalamic BP_ND_, while adjusting for age (GLM: Thalamic ΔGABA ~ thalamic BP_ND_ + age), and we evaluated if this association was different between ICB+ and ICB− patients (GLM: Thalamic ΔGABA ~ thalamic BP_ND_ + ICD status + thalamic BP_ND_*ICD status + age).

### Reporting summary

Further information on research design is available in the Nature Research Reporting Summary linked to this article.

## Supplementary information

Supplementary Figure 1

Reporting Summary Checklist

## Data Availability

The data presented in this work are available on request from the corresponding author.

## Code availability

The code used for MRS processing is available on request from the corresponding author.
