# Supplementary material for: Dopamine-induced changes to thalamic GABA concentration in impulsive Parkinson disease patients
Source: NPJ Parkinsons Dis. 2022 Apr 5;8:37. doi: 10.1038/s41531-022-00298-8 (PMC8983736; doi:10.1038/s41531-022-00298-8)
Supplement: Supplementary file 1 — Supplementary Figure 1 [file 41531_2022_298_MOESM1_ESM.pdf]

SUPPLEMENTARY MATERIAL

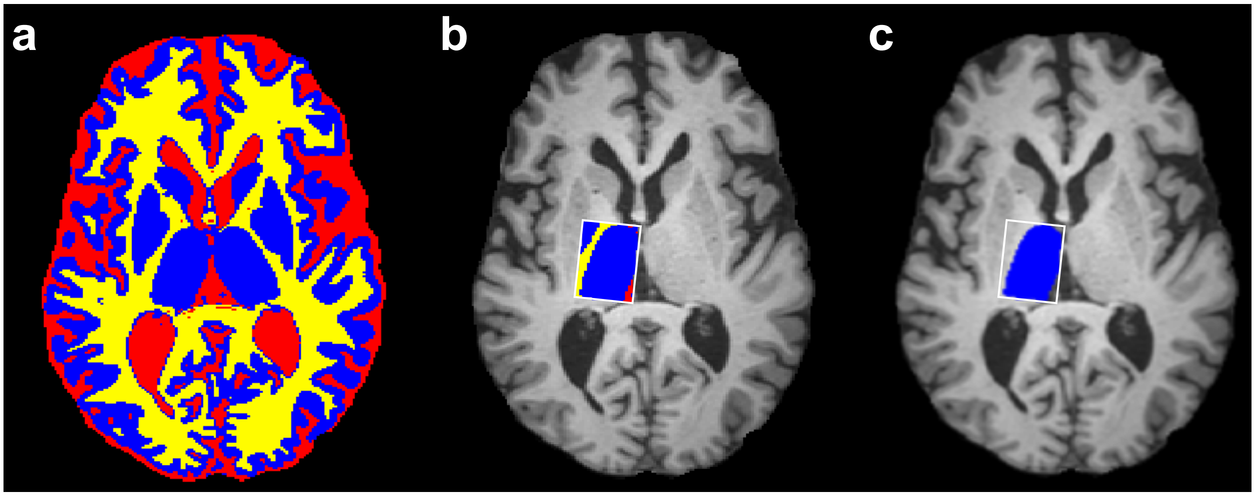

Supplementary Fig. 1 Tissue segmentation for compartment correction. **a** Grey matter (GM, blue), white matter (WM, yellow), and cerebral spinal fluid (CSF, red) tissue segmentation was obtained by merging the results from FSL FAST and FIRST. **b** The MRS voxel mask was then applied to the tissue segmentation to determine the tissue voxel fractions for GM, WM and CSF. **c** Voxel fraction corresponding to the thalamus.
