# Supplementary material for: Dopamine-induced changes to thalamic GABA concentration in impulsive Parkinson disease patients
Source: NPJ Parkinsons Dis. 2022 Apr 5;8:37. doi: 10.1038/s41531-022-00298-8 (PMC8983736; doi:10.1038/s41531-022-00298-8)
Supplement: Supplementary file 2 — Reporting Summary Checklist [file 41531_2022_298_MOESM2_ESM.pdf]

nature portfolio

Corresponding author(s): NPJPARKD-00875R

Last updated by author(s): Jan 15, 2022

Reporting Summary

Nature Portfolio wishes to improve the reproducibility of the work that we publish. This form provides structure for consistency and transparency in reporting. For further information on Nature Portfolio policies, see our Editorial Policies and the Editorial Policy Checklist.

Statistics

For all statistical analyses, confirm that the following items are present in the figure legend, table legend, main text, or Methods section.

n/a	Confirmed	
☐	☒	The exact sample size (n) for each experimental group/condition, given as a discrete number and unit of measurement
☐	☒	A statement on whether measurements were taken from distinct samples or whether the same sample was measured repeatedly
☐	☒	The statistical test(s) used AND whether they are one- or two-sided<br>*Only common tests should be described solely by name; describe more complex techniques in the Methods section.*
☐	☒	A description of all covariates tested
☐	☒	A description of any assumptions or corrections, such as tests of normality and adjustment for multiple comparisons
☐	☒	A full description of the statistical parameters including central tendency (e.g. means) or other basic estimates (e.g. regression coefficient) AND variation (e.g. standard deviation) or associated estimates of uncertainty (e.g. confidence intervals)
☒	☐	For null hypothesis testing, the test statistic (e.g. F, t, r) with confidence intervals, effect sizes, degrees of freedom and P value noted<br>*Give P values as exact values whenever suitable.*
☒	☐	For Bayesian analysis, information on the choice of priors and Markov chain Monte Carlo settings
☒	☐	For hierarchical and complex designs, identification of the appropriate level for tests and full reporting of outcomes
☒	☐	Estimates of effect sizes (e.g. Cohen's d, Pearson's r), indicating how they were calculated

Our web collection on statistics for biologists contains articles on many of the points above.

Software and code

Policy information about availability of computer code

Data collection

Data were collected on a 3T MRI scanner (Achieva, Phillips Healthcare, the Netherlands) with body coil transmit and 32-channel head coil reception. Scanning included a 3D structural T1-weighted whole brain image, (MPRAGE, TR/TE=8.9/4.6 ms; turbo gradient echo factor=131; spatial resolution=1x1x1 mm3), and single-voxel J-edited MRS using MEGA-PRESS 63 (TR/TE=3000/68 ms; 320 transients; 2048 data points at a spectra width of 2 kHz).

The Philips Vereos PET/CT is a state-of-the-art PET/CT scanner utilizing digital photon counting technology to provide 310 ps time-of-flight resolution and providing diagnostic quality CT with 64-slices.

Data analysis

MRS analysis was performed using Gannet 3.064. Frequency and phase correction and outlier rejection was applied. To account for the underlying tissue composition, we applied the alpha-correction. GannetCoRegister was used to register the MRS voxel to the T1-weighted image, and tissue segmentation was performed by merging the results obtained from FSL FAST and FSL FIRST (Supplementary Figure 1) (FSL v5.0.2.1, FMRIB, Oxford, UK).

D2-like recpetor levels were estimated using the simplified reference tissue model (SRTM) performed in PMOD software version 3.7 (PMOD Technologies, Zurich Switzerland) to measure [18F]fallypride binding potential (BPND; the ratio of specifically bound [18F]fallypride to its nondisplaceable concentration as defined under equilibrium conditions). BPND images were co-registered to the T1-weighted image using FSL FLIRT (FSL v5.0.2.1, FMRIB, Oxford, UK). SFL FIRST was used to obtain the thalamic mask and the mean BPND values were recorded.

Statistical tests were run with R version 4.1.2 R Core Team (2013). R: A language environment for statistical computing. R Foundation for Statistical Computing, Vienna Austria. URL http://www.R-project.org/.

For manuscripts utilizing custom algorithms or software that are central to the research but not yet described in published literature, software must be made available to editors and reviewers. We strongly encourage code deposition in a community repository (e.g. GitHub). See the Nature Portfolio guidelines for submitting code & software for further information.

Data

Policy information about availability of data

All manuscripts must include a data availability statement. This statement should provide the following information, where applicable:

- Accession codes, unique identifiers, or web links for publicly available datasets
- A description of any restrictions on data availability
- For clinical datasets or third party data, please ensure that the statement adheres to our policy

The data presented in this work are available on request from the corresponding author.

Field-specific reporting

Please select the one below that is the best fit for your research. If you are not sure, read the appropriate sections before making your selection.

☐ Life sciences ☒ Behavioural & social sciences ☐ Ecological, evolutionary & environmental sciences

For a reference copy of the document with all sections, see nature.com/documents/nr-reporting-summary-flat.pdf

Behavioural & social sciences study design

All studies must disclose on these points even when the disclosure is negative.

Study description	This study is a quantitative experimental study with quantitative data.
Research sample	Patients (14 without ICB, 19 with ICB) with idiopathic PD meeting UK Brain Bank criteria treated with DAA therapy were recruited from the Movement Disorders Clinic at Vanderbilt University Medical Center. In the ICB- group, there were 9 males and the average age was 67.2. In the ICB+ group, 10 were males and the average age was 61.6. As 60% of those with PD are male and the average age is 65.5, we believe these data to be representative.
Sampling strategy	Power analysis showed that a sample size of 38 patients will achieve roughly 80% power to detect a difference in means of 0.453 (the difference in GABA between pre-med mean of 1.842 and post-med mean of 2.295), assuming that the common standard deviation is 0.972 (i.e., 0.486*2, where the maximum of pre-and and post-med standard deviation is multiplied by 2 for conservative estimation) using a paired t-test with 0.05 two sided significant level.
Data collection	Patients were scanned in the Off- and On-DAA states using a 3T MRI scanner (Achieva, Phillips Healthcare, the Netherlands) with body coil transmit and 32-channel head coil reception. PET data was collected with a Philips Vereos PET/CT scanner utilizing digital photon counting technology to provide 310 ps time-of-flight resolution and providing diagnostic quality CT with 64-slices.
Timing	Data was collected from 2015-2020.
Data exclusions	No data was excluded from analysis.
Non-participation	While 33 participants completed the study, only a subset (20 participants) completed the PET protocol. This was due to concerns surrounding radiation exposure, participant retention, and motor symptoms in the off-medication state.
Randomization	All participants completed the OFF and ON study arms.

Reporting for specific materials, systems and methods

We require information from authors about some types of materials, experimental systems and methods used in many studies. Here, indicate whether each material, system or method listed is relevant to your study. If you are not sure if a list item applies to your research, read the appropriate section before selecting a response.

Materials & experimental systems

n/a	Involved in the study	
☒	☐	Antibodies
☒	☐	Eukaryotic cell lines
☒	☐	Palaeontology and archaeology
☒	☐	Animals and other organisms
☐	☒	Human research participants
☒	☐	Clinical data
☒	☐	Dual use research of concern

Methods

n/a	Involved in the study	
☒	☐	ChIP-seq
☒	☐	Flow cytometry
☐	☒	MRI-based neuroimaging

Human research participants

Policy information about studies involving human research participants

Population characteristics	Participants with idiopathic Parkinson's Disease were included in this study. All subjects were age and gender matched. The population was dichotomized into ICB positive and negative groups.
Recruitment	Participants were recruited from the Vanderbilt University Medical Center Movement Disorder clinic.
Ethics oversight	The Vanderbilt University Institutional Review Board reviewed and approved this study (#151908, #160213).

Note that full information on the approval of the study protocol must also be provided in the manuscript.

Magnetic resonance imaging

Experimental design

Design type	Resting State
Design specifications	Two MRI scans were performed per subject (on and off DAA). One PET scan was performed per subject.
Behavioral performance measures	No behavioral data was collected.

Acquisition

Imaging type(s)	Structural and MRS
Field strength	3T
Sequence & imaging parameters	Scanning included a 3D structural T1-weighted whole brain image, (MPRAGE, TR/TE=8.9/4.6 ms; turbo gradient echo factor=131; spatial resolution=1x1x1 mm3), and single-voxel J-edited MRS using MEGA-PRESS 63 (TR/TE=3000/68 ms; 320 transients; 2048 data points at a spectra width of 2 kHz). The spectroscopy voxels were planned off orthogonal reconstructions of the high-resolution T1-weighted scan and placed in the right thalamic area (voxel dimensions=30x22x28 mm3) (Figure 1a) and the right motor cortex (voxel dimensions 40x25x25 mms) (Figure 1b). Editing pulses (14 ms, 140 Hz bandwidth) were applied at 1.9 ppm and 8 ppm on alternate scans. An unedited MRS scan without water suppression was also acquired for normalization.
Area of acquisition	Right motor cortex and thalamus were used as regions of interest in this study.

Diffusion MRI ☐ Used ☒ Not used

Preprocessing

Preprocessing software	To account for the underlying tissue composition, we applied the alpha-correction. GannetCoRegister was used to register the MRS voxel to the T1-weighted image, and tissue segmentation was performed by merging the results obtained from FSL FAST and FSL FIRST (Supplementary Fig. 1) (FSL v5.0.2.1, FMRIB, Oxford, UK). The MRS voxel mask was then applied to the tissue segmentation to determine the tissue voxel fractions for gray matter (GM), white matter (WM) and cerebrospinal fluid (CSF) (Supplementary Fig. 1). The compartment correction (using alpha=0.5, Wanasapura values for relaxation parameters, and 36.1 mol/dm3, 43.3 mol/dm3, and 53.8 mol/ dm3 for MR-visible water concentrations for WM, GM, and CSF, respectively), and tissue normalization were applied to account for differences in GABA+/water concentration between GM and WM, and to obtain the compartment corrected thalamic and motor cortex GABA+ concentration. A similar correction was applied to the Glx/water measures to obtain the compartment corrected Glx concentration.
Normalization	All image analyses were performed in subject space.
Normalization template	N/A
Noise and artifact removal	The compartment correction (using alpha=0.5, Wanasapura values for relaxation parameters, and 36.1 mol/dm3, 43.3 mol/dm3, and 53.8 mol/ dm3 for MR-visible water concentrations for WM, GM, and CSF, respectively), and tissue normalization were applied to account for differences in GABA+/water concentration between GM and WM, and to obtain the compartment corrected thalamic and motor cortex GABA+ concentration. A similar correction was applied to the Glx/water measures to obtain the compartment corrected Glx concentration.
Volume censoring	N/A

Statistical modeling & inference

Model type and settings	To understand whether thalamic GABA changes are different between ICB+ and ICB-, we performed a general linear regression model (GLM) analysis specifying thalamic ΔGABA as dependent variable, ICB status as independent variable, and age and DAA dosage (i.e. agonist single dose equivalent) as covariates (GLM: Thalamic ΔGABA ~ ICD status + age + DAA

dosage). To evaluate if thalamic GABA changes were related to a quantitative marker of impulsivity, we performed GLM analyses specifying ΔGABA as dependent variable, QUIP-RS score as independent variable, and age and DAA dosage as covariates (GLM: Thalamic ΔGABA ~ QUIP-RS + age + DAA dosage). The above GLM analyses were also performed for the motor cortex GABA and the thalamic glutamate as dependent variables. Finally, we tested the association between the changes in thalamic GABA and the thalamic BPND, while adjusting for age (GLM: Thalamic ΔGABA ~ thalamic BPND + age), and we evaluated if this association was different between ICB+ and ICB- patients (GLM: Thalamic ΔGABA ~ thalamic BPND + ICD status + thalamic BPND*ICD status + age).

Effect(s) tested

To understand whether thalamic GABA changes are different between ICB+ and ICB-, we performed a general linear regression model (GLM) analysis specifying thalamic ΔGABA as dependent variable, ICB status as independent variable, and age and DAA dosage (i.e. agonist single dose equivalent) as covariates (GLM: Thalamic ΔGABA ~ ICD status + age + DAA dosage). To evaluate if thalamic GABA changes were related to a quantitative marker of impulsivity, we performed GLM analyses specifying ΔGABA as dependent variable, QUIP-RS score as independent variable, and age and DAA dosage as covariates (GLM: Thalamic ΔGABA ~ QUIP-RS + age + DAA dosage). The above GLM analyses were also performed for the motor cortex GABA and the thalamic glutamate as dependent variables. Finally, we tested the association between the changes in thalamic GABA and the thalamic BPND, while adjusting for age (GLM: Thalamic ΔGABA ~ thalamic BPND + age), and we evaluated if this association was different between ICB+ and ICB- patients (GLM: Thalamic ΔGABA ~ thalamic BPND + ICD status + thalamic BPND*ICD status + age).

Specify type of analysis: ☐ Whole brain ☒ ROI-based ☐ Both

Anatomical location(s): The right motor cortex and thalamus were used.

Statistic type for inference (See Eklund et al. 2016)

GLM, Wilcoxon Rank Sum, and Chi Squared Test.

Correction

No correction was used.

Models & analysis

n/a	Involved in the study
☒	☐ Functional and/or effective connectivity
☒	☐ Graph analysis
☒	☐ Multivariate modeling or predictive analysis
